# The complete mitochondrial genome of a whiter-spotted flower chafer, *Protaetia brevitarsis* (Coleoptera: Scarabaeidae)

**DOI:** 10.1080/23802359.2020.1824592

**Published:** 2020-10-21

**Authors:** Eun Hwa Choi, Sohyun Mun, Su Youn Baek, Jihye Hwang, Ui Wook Hwang

**Affiliations:** a Department of Biology Education, Teachers College & Institute for Phylogenomics and Evolution, Kyungpook National University, Daegu, Republic of Korea; b Institute for Korean Herb-Bio Convergence Promotion, Kyungpook National University, Daegu, Republic of Korea

**Keywords:** *Protaetia brevitarsis*, Coleoptera, mitochondrial genome, phylogenetic analysis

## Abstract

The complete mitochondrial genome of *Protaetia brevitarsis,* an important Scarabaeidae insect that is distributed across most Asian countries, was characterized using long template PCR methods. It was 17,783 bp in length being composed of 13 protein coding genes (PCGs), 22 transfer RNA genes (tRNAs), two ribosomal RNA genes (rRNAs) and a non-coding region. The phylogenetic tree reconstructed based on the maximum likelihood (ML) method confirmed that *P. brevitarsis* was placed within the clade of Scarabaeidae and Polyphaga species forming a complete monophyly.

*Protaetia brevitarsis seulensis* (Coleoptera: Scarabaeidae: Cetoniinae), commonly known as a white-spotted flower chafer, has long been used medicinal resources for liver‐related disease prevention and anticancer effects in Korea (Kwon 2009). Moreover, *P. brevitarsis* larvae is considered to be available as a future food resource and discussions on commercialization through mass breeding have recently been made (Adámková et al. 2017). However, *P. brevitarsis* is still unknown, both genetically and evolutionarily, and thus stepwise investigation and data accumulation about this species are required prior to commercialization. In this study, we provide the complete mitochondrial genome of this species (GenBank accession no. MN418316) to provide clear understanding of its phylogenetic placement in coleopterans and genetic markers that can be useful in industrial purposes.

*P. brevitarsis* individual used for our genetic analysis was collected from Cheongdo IRUDA21, Cheongdo-gun, Gyeongsangnam-do, South Korea (35°38′07.9″N 128°38′06.7″E). The specimen was deposited in the Institute for Phylogenomics and Evolution, Kyungpook National University in South Korea under the voucher number PB2019001H. DNA was extracted using QIAamp Tissue Kit (Qiagen, Valencia, CA) and the mitochondrial genome was characterized by primer-walking using long template PCR products (Roche, co. Germany). Sequences were aligned and trimmed using the Clustal W program in BioEdit 7.0.9 (Hall 1999). Protein coding genes (PCGs), rRNAs, tRNAs and D-loop were confirmed using NCBI Basic Local Alignment Search Tool (BLAST) (Altschul et al. 1990) and tRNA-scan 1.21 (Lowe and Eddy 1997).

The complete mitochondrial genome of *P. brevitarsis* was 17,783 bp in length being composed of 13 PCGs, 22 tRNAs, 2 rRNAs and a non-coding region. The gene arrangement of the mitochondrial genome of *P. brevitarsis* is similar to other related beetle species (Arnoldi et al. 2007, Kim et al. 2012, Kim et al. 2014). The genome was asymmetric in nucleotide composition with a strong AT bias (75.1% A + T content). *Nad5, nad4, nad4L, nad1, rrnL, rrnS, trnF, trnH, trnP, trnL, trnV, trnG, trnY and trnC* were located on heavy strand, whereas *nad3, cox3, atp6, atp8, cox2, cox1, nad2, cob, nad6, trnQ, trnS, trnN, trnR, trnA, trnG, trnD, trnK, trnW, trnL, trnM, trnI* and *trnS* on the light strand. The mitochondrial genome of *P. brevitarsis* contained a species-specific noncoding sequence (3,120 bp in length) between *rrnS* and *trnI* that have been found in the previous study (5,654 bp in length; Kim et al 2014).

The maximum likelihood (ML) tree was reconstructed using 13 PCGs to investigate the taxonomic position of *P. brevitarsis* in coleopterans. The result confirmed that *P. brevitarsis* was placed in the clade of scarabaeids and the monophyly of Polyphaga clade was well-supported. In addition, Cucujiformia, Elateriformia, Scirtiformia and Scarabaeiformia formed clear monophyletic groups, respectively (Figure 1). The relationships among the major infraorders of Coleoptera are similar to those of Yuan et al. (2016), except for the phylogenetic position of Adephage and Myxophaga.

**Figure 1.**
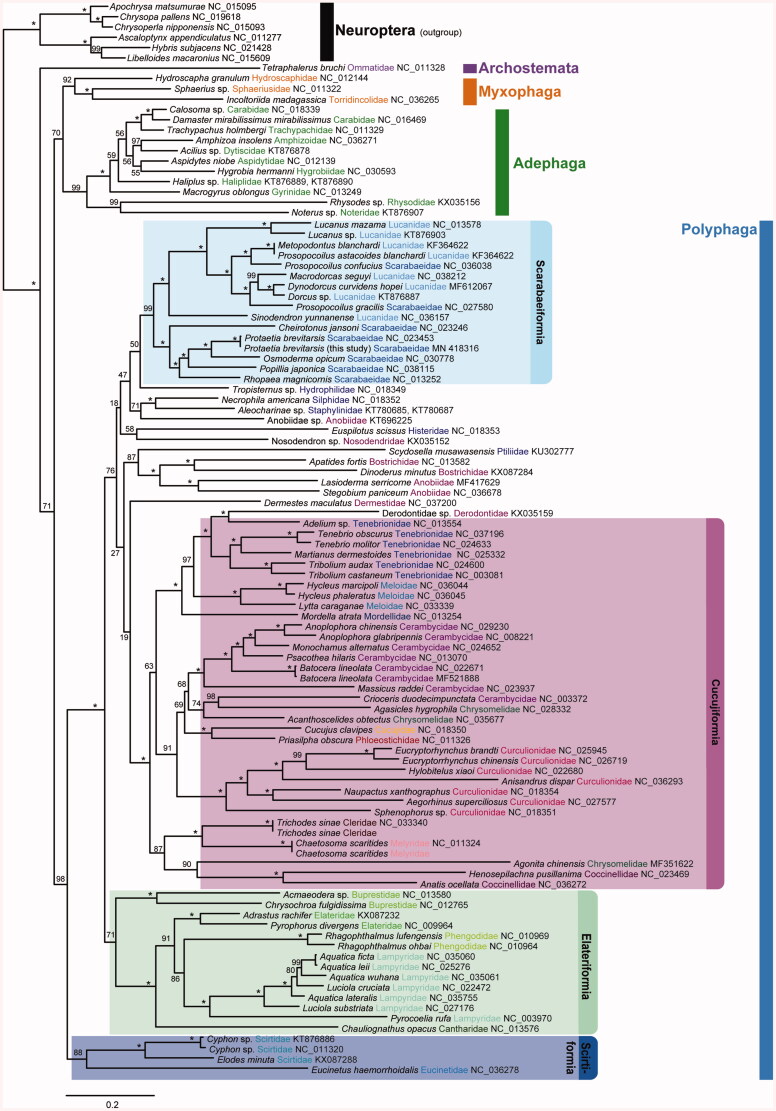
Mitochondrial genomic phylogeny reconstructed by ML method using 13 protein coding gene. The confidence of branch supports was shown in number inferred from ultrafast bootstrap method using IQ-TREE.

## Data Availability

The data that support the findings of this study are openly available in NCBI at https://www.ncbi.nlm.nih.gov/nuccore/MN418316, reference number MN418316.
